# Performance Evaluations of PC5-Based Cellular-V2X Mode 4 for Feasibility Analysis of Driver Assistance Systems with Crash Warning

**DOI:** 10.3390/s20102950

**Published:** 2020-05-22

**Authors:** Takeshi Hirai, Tutomu Murase

**Affiliations:** 1 Graduate School of Informatics, Nagoya University, Nagoya 464-8601, Japan; 2 Information Technology Center, Nagoya University, Nagoya 464-8601, Japan; tom@itc.nagoya-u.ac.jp

**Keywords:** PC5-based C-V2X mode 4, sensing-based semi-persistent scheduling, driver assistance system, crash warning

## Abstract

This paper presents the communication performance of PC5-based Cellular-Vehicle-to-Everything mode 4 (called mode 4) to measure the feasibility of a Crash Warning System (called CWS). The CWS requires nodes (e.g., cars or pedestrians) to update its location information ten times per second. This requirement provides us with a channel congestion problem. To investigate feasibility in terms of channel congestion, we evaluated communication performance in various crash scenarios by computer simulation. One of the serious scenarios is a crowded environment like large intersections. In the uniform node distribution, in which we evaluated average performance, mode 4 accommodated 26% fewer nodes than the expected one; in a realistic node distribution, mode 4 achieved 55% worse performance than the CWS requirements. Our results highlighted the need for performance improvements of mode 4 for CWS in practical uses.

## 1. Introduction

In order to advance the Intelligent Transport Systems (ITS) concept for Smart Cities [1], driver assistance systems require Vehicle-to-Everything (V2X) communications. In particular, the Crash Warning System (CWS) [2], one of the essential systems using V2X, improves the safety level of driving. In this system, each communication node, simply called a node, e.g., a vehicle, a pedestrian, or a drone with communication equipment, periodically advertises its state data on its frames (simply called frames) in the broadcast mode. CWS requires reliable V2X communication, i.e., with severe Quality of Service (QoS) requirements, to accurately warn users. Of these requirements [3,4], nodes must receive status data from other nodes at rates of ten or more times per second. Additionally, nodes must warn users at least 2.5 s prior to future traffic accidents, i.e., potential crashes. Based on the QoS requirements, we have defined a performance metric called *Node Accommodation Capacity* (NAC), which represents the maximum number of nodes that can satisfy the QoS requirements. This metric plays an important role in proving the feasibility. We require enough large NACs to use CWS in various environments [5].

As a new V2X standard, PC5-based Cellular-V2X (C-V2X) mode 4 (simply called mode 4) [6] has been developed. Mode 4 does not require communications with base stations. This characteristic provides device-to-device communications, even out of base stations’ coverage range. It also reduces the communication costs with base stations. Instead of communications with base stations, mode 4 has a new random access mechanism—sensing-based semi-persistent scheduling (Sensing-based SPS). This random access mechanism uses a transmission history of the past 1000 milliseconds. The history supports nodes to select a slot with estimated-low interference. The new standard supports CWS, and thus, this paper focuses on CWS using mode 4.

The congestion problems of mode 4 disturb the development of CWS in terms of practical uses. In crash scenarios, mode 4 requires reliable communications. Unfortunately, some crash scenarios decrease the signal-to-interference-plus-noise ratio (SINR); for example, in crowded intersections or for high-speed vehicles like emergency vehicles, mode 4 experiences lower SINR than other scenarios. In such scenarios, mode 4 may not satisfy the QoS requirements. However, existing works have not evaluated the performance of mode 4 in crash scenarios. In such crash scenarios, we need to focus on nodes approaching to collide with the other nodes after a few seconds. The movements vary the SINRs between the nodes. Most of the works, like [7,8,9,10,11,12,13,14,15,16], did not focus on CWS and thus did not consider the crash scenarios for CWS. Our previous works [17,18,19] evaluated performance under congestion in CWS, but the nodes were fixed. Thus, these works also did not consider crash scenarios sufficiently.

In this paper, we investigate the feasibility of CWS in terms of the congestion caused by crash scenarios. In the end, we model crash scenarios in crowded intersections or for high-speed vehicles, and evaluate the performance characteristics of mode 4 in the modeled scenarios. The former scenarios are mainly related to the density of the nodes. By using the uniform node distribution, we investigate the performance limitations of mode 4 against the number of nodes, i.e., NAC. Additionally, by using realistic node distributions, we investigate the performance characteristics of mode 4 in realistic intersections. The latter scenarios depend on the relative speed of two nodes related to a potential crash. We evaluate the performance limitations of mode 4 at various relative speeds. In summary, this paper mainly contributes to investigating the NAC of mode 4 in various crash scenarios in CWS in order to show the feasibility in congestion conditions.

## 2. Related Works

Table 1 summarizes the existing works related to mode 4 [7,8,9,10,11,12,13,14,15,16,17,18,19]. Some works [7,8,9,10,11,12,13,14,15,16] evaluated the performance of PHY, MAC, or the application (such as cooperative awareness and platoon) layers of mode 4 rather than the performance in use-cases of CWS using mode 4. Our previous works [17,18,19] focused on the performance of mode 4 in CWS but did not cover feasibility. In the following paragraphs, we briefly explain the related works.

Some related works have focused on the performance of mode 4 in the PHY and MAC layers. The work in [7] summarized the mechanism of mode 4. In [7], the authors evaluated the packet delivery rates of the distance between a transmitter and a receiver. The study in [8] simulated the performance of some mode 4 configuration parameters for slot selection. In [9], the authors mathematically analyzed the performance of mode 4 in terms of the error and the distance between a transmitter and a receiver. Reference [10] simulated the performance of mode 4 for frame sizes. In [11,12], the authors also simulated the performance of mode 4 for its key configuration parameters. The work in [13] enhanced the mechanisms of mode 4 to reduce collision errors by slot reselections.

The other works in [14,15,16,17,18,19] evaluated the performance of mode 4 while considering the application layer. In [14,15], the authors evaluated the performance of mode 4 in terms of cooperative awareness. Paper [14] revealed that mode 4 is characterized by greater delay and less packet delivery rates than those of IEEE802.11p. The work in [15] concluded that it might be possible to utilize mode 4 for practical uses in terms of cooperative awareness by optimizing the modulation and coding scheme (MCS). In [16], the authors simulated the performance of mode 4 in the use cases of the platoon, and concluded that mode 4 outperforms IEEE802.11p in the use cases. The work considers traffic accidents in terms of the platoon. However, the relative speed between platooned trucks is smaller than that in CWS. Our previous works [17,18,19] focused on CWS using mode 4. In [17,19], we proposed the non-orthogonal multiple access (NOMA) concept to overcome the congestion problem of mode 4. In [18], we investigated the behaviors of the slot selection mechanisms of mode 4. Our previous works did not however cover investigations into the feasibility of mode 4 in various crash scenarios, because the previous works focused on comparing communication systems rather than the feasibility evaluations of mode 4 in CWS.

According to the above discussions, the existing works have not investigated the feasibility of mode 4 in CWS. In this paper, we model various crash scenarios and investigate the performance of mode 4 in the modeled crash scenarios. The investigations highlight the performance limitations of mode 4 in CWS. Therefore, this paper extends the fundamental performance evaluations to the feasibility investigations for CWS.

## 3. System Model and Characteristics

This section explains the procedure of mode 4, the model of CWS, and the characteristics of mode 4 in CWS. In the model of CWS, we describe the warning process of CWS and the QoS requirements for accurate warnings of CWS. Explaining the characteristics help to understand our simulation results in Section 5.

### 3.1. Mode 4 Mechanism

**Figure 1:**
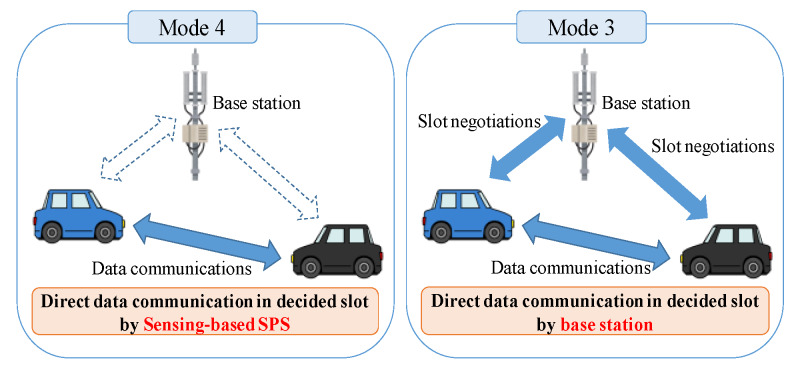
The characteristics of mode 4, as compared to mode 3.

Mode 4 does not require any base stations to communicate with the other nodes, unlike other C-V2X standards such as Uu-based C-V2X and PC5-based C-V2X mode 3 (called mode 3) [20]. In the Uu-based C-V2X, each node must negotiate the transmission slots with a base station before transmissions. Subsequently, the node transmits data to the base station once, and then the base station transmits the data to the other nodes. In contrast, mode 3 and mode 4 use PC5 interfaces to transmit data, i.e., sidelinks. Figure 1 shows the comparison between the characteristics of mode 4 and mode 3. Mode 3, the right figure in Figure 1, requires negotiation for a transmission slot, but directly transmits data through sidelink. In mode 4, the left figure in Figure 1, each node autonomously selects a transmission slot and directly transmits data in the slot [6]. In summary, mode 4 has no additional infrastructures and no control signals, unlike mode 3; it reduces communication costs, as compared to the other C-V2X standards. We explain the algorithm of mode 4 as follows.

In order to communicate without base stations, each node executes Sensing-based SPS, which is a new random-access protocol. Sensing-based SPS has two different features from CSMA/CA as follows:

⋅ Slot selection based on history: In order to select a good slot for the transmissions, each node uses the history of past slot utilization and estimates the interference of each future slot. The history includes the sensing information of all the slots, despite it transmitting or not; that is, each node senses all the slots. This sensing information includes the received signal, the remaining number of slot utilization [7] (related to the next feature), and others.
⋅ Semi-persistent slot utilization: In order to boost the above estimation accuracy of the interference pattern, each node uses the same slot in a semi-persistent manner; in other words, each node successively uses the same frequency resource at specific times.

**Figure 2:**
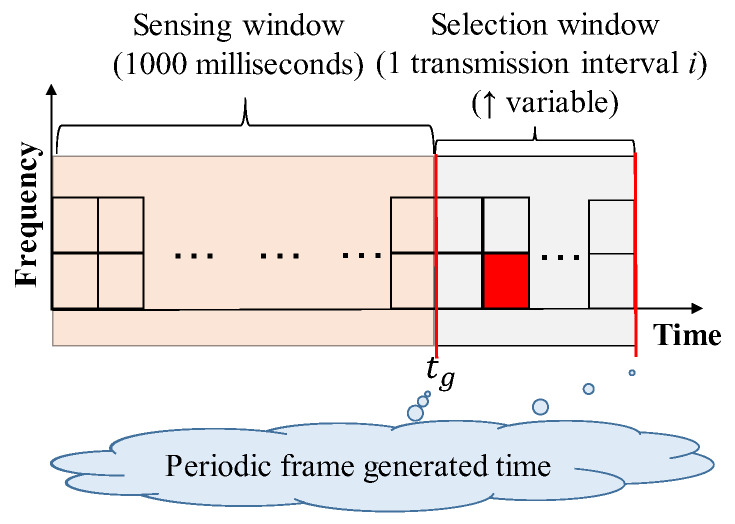
The illustration of the sensing window and selection window.

The basic procedure of the Sensing-based SPS has seven steps. The first four steps are to reduce the candidate slots using some carrier sensing mechanisms (Step 1–4). Specifically, each node filters the estimated slots with high interference in Steps 2–4. The last three steps operate successive transmissions (Step 5–7). We describe the steps in the following paragraphs:

Step 1. Initialization: When a frame is generated, each node initializes a set of candidate slots, $S_{A}$. The initial set includes all the slots during the *selection window*—as shown in the right part of Figure 2. The selection window is during a transmission interval *i*, e.g., 100 ms for 10 frames/s, which is after its generated time $t_{g}$ of the periodic frames. In contrast, the sensing history includes all the slots during the sensing window—as shown in the left part of Figure 2. As shown in Figure 2, the length of this window is standardized at 1000 ms.

Step 2. Filtering 1 (half-duplex): In this step, each node excludes non-sensed candidate slots during the sensing window from the initial set $S_{A}$. For example, each node cannot receive frames in the transmission slot. In these non-sensed slots, nodes cannot infer the slot usage from the past slots due to the half-duplex. The updated $S_{A}$ passes to the next step.

Step 3. Filtering 2 (Reference Signal Received Power (RSRP)): In this step, each node excludes specific candidate slots by the RSRP conditions from $S_{A}$. The RSRP conditions are as follows;

– The first condition is that one or more nodes reserve the candidate slot in the sensing window. Each node refers to the remaining number of sensing information in the past slot. Note that the frame must be decoded successfully.
– The second condition is that the RSRP of the above slots, reserved by the other nodes, is higher than the threshold. Note that, when the same transmitter reserves a transmission slot, the RSRP of the most recent slot is referred to.

**Figure 3:**
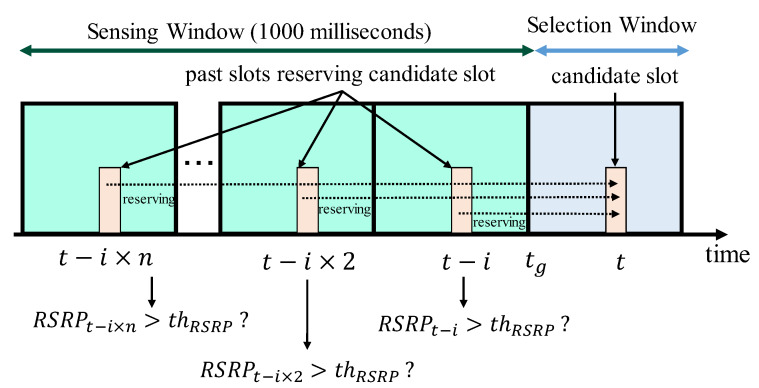
The abstract image of the RSRP mechanisms at time $t_{g}$.

From these two conditions, each node can exclude the candidate slots with deterministically high interference in this step. Figure 3 illustrates an example of Step 3. In Figure 3, in the three past slots, the nodes reserve a candidate slot of time *t*. This step is iterated until the size of $S_{A}$ becomes 20% or higher of the initial size in order to ensure that a sufficient amount of candidate slots are handed over to the next step. With each iteration, the RSRP threshold is reduced by 3 dB of the standard.

**Figure 4:**
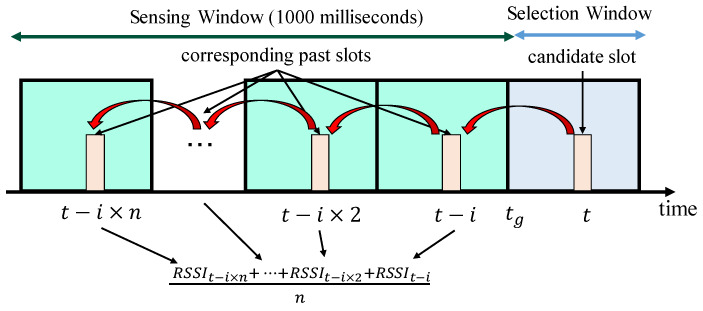
The abstract image of the RSSI mechanism at time $t_{g}$.

Step 4. Filtering 3 (Received Signal Strength Indicator (RSSI)): Each node excludes the candidate slots based on the average RSSI over the corresponding past slots. The corresponding past slots are defined as the slots that are traced back with the transmission interval from the candidate slot in the sensing window. Figure 4 is an example of a node calculating the average RSSI. Then, an individual node puts the *N* top of the slots with the lowest average RSSI into the new set ($S_{B}$). The *N* is standardized as the value of 20% of the number of slots. This step estimates the interference, although the nodes cannot fully identify the interference. In other words, the nodes exclude the candidate slots with the estimated-high interference.

Step 5. Random Selection: Each node randomly selects a transmission slot from $S_{B}$. This random selection prevents several nodes from selecting the same slot.

Step 6. Configuration: Each node randomly sets a reselection counter from a pre-defined range. Nodes reduce the counter by one every transmission. The node successively uses the same slot selected in Step 5 until the reselection counter becomes zero. The random selection of the reselection counters is effective in avoiding simultaneous reselections with other nodes. As a result, the possibility of the node selecting the same slot with other nodes is decreased.

Step 7. Successive transmissions and Reselection: Each node successively uses the selected slot and probabilistically reselects the next transmission slot after the reselection counter becomes zero. The node returns to Step 1 at a probability, called resource keeping probability. The probability is pre-defined in the range of 0–0.8; otherwise, each node maintains and uses the same slot.

### 3.2. Crash Warning System

#### 3.2.1. How to Warn

**Figure 5:**
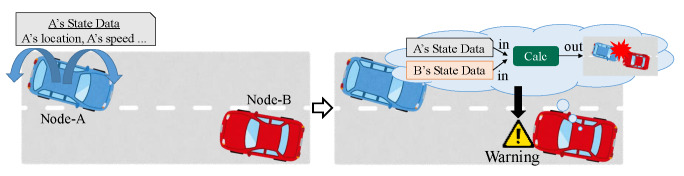
The procedures of CWS: broadcast to surrounding nodes and warn the users.

In CWS, each node advertises (broadcasts) its own state data periodically and calculates the crash risks in the near future (i.e., potential crash risks), based on the obtained state data from other nodes. Figure 5 shows an example of a warning in CWS. The left illustration shows a periodical advertisement phase, and the right illustration shows a calculation phase.

In the periodical advertisement phase, each node broadcasts a frame, including its own state data in every transmission period. Each data includes the node’s location, speed, direction, time, and other information related to the node’s mobility. The data can be obtained from some sensors, such as GPSs and gyro sensors. In Figure 5, node-A broadcasts its own state data to notify other nodes of its existence.

In the calculation phase, CWS calculates the user’s potential crash risks in every receiving frame. If the risks are higher than a pre-defined value, the function warns the user, such as the driver or pedestrian. In Figure 5, node-B detects the potential crash risks with node-A from the obtained frame, and thus, warns the driver.

#### 3.2.2. QoS Requirements

CWS has the following two QoS requirements to detect *potential crash nodes*, which are nodes that are likely to collide with other nodes.

**Figure 6:**
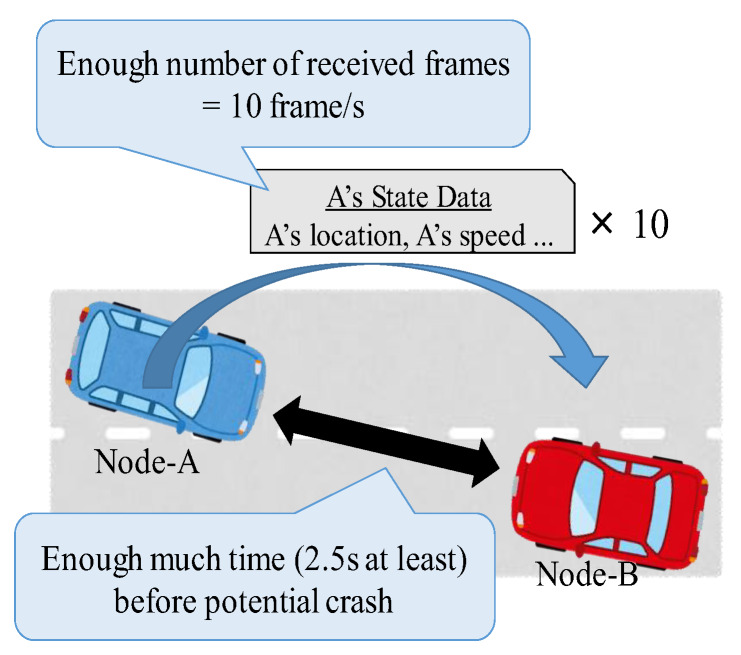
An abstract image of the QoS requirements of CWS for node-B to detect node-A.

Figure 6 illustrates an outline of the QoS requirements for node-B to detect node-A.

⋅ *The number of received frames*: Potential crash nodes must receive ten frames/s from the corresponding potential crash nodes. Reference [3] standardizes the number of transmissions to 10 frames/s. We interpret this specification as a requirement concerning the number of received frames because receiving all of the transmitted frames is typically necessary and important in warning of potential crashes; in other words, the nodes need to obtain enough information to predict their future location in a real-time manner accurately. In Figure 6, node-B needs to receive at least ten numbers of node-A’s frames per second.
⋅ *Warning period*: This is the period during which the system can safely warn the user. Specifically, this period is during 2.5–9.5 s prior to potential crashes [4]; in other words, the time-to-crash is within 2.5–9.5 s. To warn at a time-to-crash of 2.5 s, which is the last warning opportunity, potential crash nodes must satisfy the above requirement during the time-to-crash of 2.5–3.5 s. In Figure 6, node-B needs to warn node-A during this period.

In summary, according to the two QoS requirements, each potential crash node must receive ten frames from the corresponding potential crash nodes during 2.5–3.5 s before the potential crashes. As aforementioned, NAC is the maximum number of nodes in the communication range when the potential crash nodes can satisfy the two requirements. On average, the system can warn accurately when the number of nodes in the communication range is equal to or less than NAC. It is necessary to increase the NAC related to various factors, such as node density.

### 3.3. Crash Scenarios and Congestion Problem

In this section, we analyze the performance of mode 4 in some crash scenarios. The following scenarios decrease communication performance;

– Crowded intersection scenarios: crashes in crowded intersections.
– High-speed scenarios: crashes with high-speed nodes, like emergency vehicles.

To discuss the relationships between the performance of mode 4 and the scenarios, we consider an SINR during the warning period between two potential crash nodes: a transmitter *t* and a receiver *r*. Equation (1) denotes the SINR $\gamma_{r}$:

(1) $$\gamma_{r}=\frac{S_{tr}}{I_{r}+N_{0}}$$

$N_{0}$ is noise power. $I_{r}$ is the interference power against the received signal at the receiver *r*. $S_{tr}$ is the received signal power from *t* to *r*. The received signal power is formulated as follows:

(2) $$S_{tr}=P_{t}C_{tr}d_{tr}{}^{-\alpha }\;\;\;$$

$P_{t}$ is the constant transmission power. $d_{tr}$ is the distance between *t* and *r*. $C_{tr}$ is the fading gain between *t* and *r*. α denotes the path loss exponent. In the following sub-sections, we discuss the performance characteristics of mode 4 in the two scenarios.

#### 3.3.1. Crowded Intersection Scenarios

Crowded intersections amplify the interference from other nodes. The interference is formulated as follows:

(3) $$I_{r}=\underset{i\in ℐ}{\sum }S_{ir}$$

ℐ denotes the set of other transmitters at the transmission opportunity of the transmitter *t*. |ℐ| is the number of simultaneous transmitters. From Equations (2) and (3) is transformed as follows:

(4) $$I_{r}=\underset{i\in ℐ}{\sum }P_{t}C_{ir}d_{ir}{}^{-\alpha }$$

The SINR in Equation (1) is transformed from Equation (4) as follows:

(5) $$\gamma_{r}=\frac{S_{tr}}{\sum_{i\in ℐ}P_{t}C_{ir}d_{ir}{}^{-\alpha }+N_{0}}$$

From Equation (5), as $d_{ir}$ is smaller and/or |ℐ| is larger, SINR is smaller. In crowded intersections, i.e., the density of the nodes is high, the number of nodes near the transmitter is, on average, larger than in non-crowded intersections. The trends of $d_{ir}$ and/or |ℐ| decrease SINR, and as a result, the performance of mode 4 is downgraded in such intersections.

Additionally, |ℐ| is related to the communication traffic load. Thus, the load is related to the number of transmissions per second (defined as NTS) is related to |ℐ|, in addition to the density of the nodes involved in the crash scenarios. Increasing NTS increases |ℐ|. Whereas, increasing NTS is expected to improve SINR because of the redundant transmissions. The two impacts also operate the degree of congestion.

#### 3.3.2. High-Speed Scenarios

In high-speed crash scenarios, $S_{tr}$ is weaker than in low-speed scenarios. In the high-speed scenarios, potential crash nodes approach each other at high speeds. As nodes move at higher speeds, the relative speed $v_{tr}$ is higher. For example, emergency vehicles run at high-speeds. By using time-to-crash $t_{crash}$ and the relative speed, the communication distance during the warning period is shown as follows:

(6) $$d_{tr}=v_{tr}t_{crash}$$

From Equations (2) and (6), the SINR is formulated as follows:

(7) $$\gamma_{r}=\frac{P_{t}C_{tr}{\left(v_{tr}t_{crash}\right)}^{-\alpha }}{I_{r}+N_{0}}$$

In order to recognize the potential crashes, the nodes need to receive frames during the warning period, i.e., $t_{crash}$ is 2.5–3.5 s at least. As the relative speed is larger at time $t_{crash}$, the communication distance is larger. As a result, the SINR is lower than that of the low-speed scenarios.

## 4. Evaluation Model

### 4.1. Crash Scenarios and Performance Metrics

**Figure 7:**
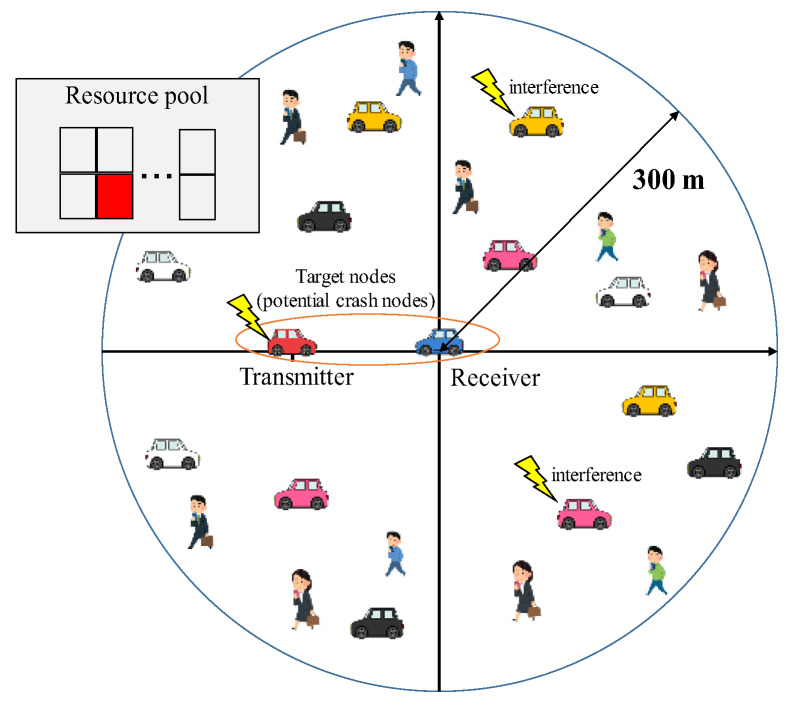
The abstract image of the simulation model of CWS: the transmitter and receiver are potential crash nodes.

Figure 7 depicts an example of the simulations. We investigated the number of received frames during the warning period at an evaluation target receiver from an evaluation target transmitter. The two target nodes were the potential crash nodes for each other; specifically, they frontally approached each other at a relative speed $v_{tr}$. This head-on crash scenario is the largest relative speed of the other scenarios, and thus, the communication distance is the largest in Equation (6). If the QoS requirements are satisfied in this scenario, the requirements are also satisfied in the other scenarios.

Around the target receiver, within 300 m, the other nodes were deployed, as shown in Figure 7. All the nodes, including target nodes, shared spectrum resources in a multiple access manner. The manner causes the other nodes to play a role in the interference and competitors of slot selections for target nodes. Figure 7 shows an example in which two nodes interfere with a frame from the target transmitter at the target receiver. The signal power was strong enough at the target receiver within 300 m.

Next, we explain the dynamic ranges of the parameters modeling the crash scenarios. For crowded intersection scenarios, two kinds of densities (i.e., node distribution models) were given. The first kind of model is the uniform distribution model. This model is the most fundamental stochastic model. The model provides us with the average performance. Additionally, the stochastic model allows us to change the number of nodes in order to boost the density of the nodes. The two reasons provide an average upper limit of the number of nodes, i.e., NAC. The second one is the realistic distribution model. The model is built by Bologna data [21], which is the realistic mobility data for cars and pedestrians. From Bologna data, we modeled three intersections: large, medium, and small intersections, named A–C, respectively. The next section explains the details of the data. The NTS of each node depends on the degree of the congestion. In our simulations, NTS was also given 10–30 frames/s. Considering high-speed scenarios, we set $v_{tr}$ at 120–240 km/h. For the scenarios, we assumed that emergency vehicles run at high speeds in urban areas. In our models, the target nodes approach at a given relative speed.

This paper showed the number of received frames during 2.5–3.5 s and NAC as a performance metric. The first metric was compared to the QoS requirements. For one of the references of NAC, we computed the *average number of nodes (ANN)*, which is an average value over the number of nodes used in the three realistic intersections from Bologna data. ANN had 488 nodes. The number of received frames were averaged over 300 iterations in the uniform node distribution model and over 3 iterations × 1 period for the traffic signal. The period of A is 105 s. B and C have no traffic signals, so we defined 112 s as the period because the duration is averaged over other traffic signals in the data. The metric was simulated by a simulator that we made. In our investigations, we could not find suitable mode 4 simulators that were able to evaluate the system-level performance in the context of CWS. We confirmed that the simulator outputted values along with the characteristics of mode 4.

### 4.2. Bologna Data and Realistic Node Distribution Models

Bologna data is the open data in iTETRIS [22] (http://sumo.dlr.de/wiki/Data/Scenarios). The Bologna data was collected in Bologna (Italy) over three days from November 11 (Tue) to November 13 (Thu) in 2008 with 636 detectors.

**Figure 8:**
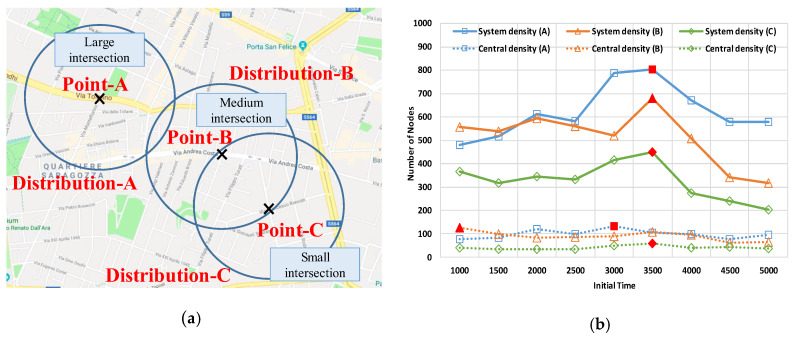
The three extracted intersections (A—C) and the statistic information about system density and central density. (**a**) Location and size of the three intersections (A—C); (**b**) The system density and central density in three intersections (A—C).

The data includes peak hours in the morning. This data includes mobility data of pedestrians. The data is suitable to evaluate V2X performance, including Vehicle-to-Pedestrian. We obtained the mobility data from the Bologna data by Simulation of Urban MObility (SUMO) [23]. From the Bologna data, we extracted three intersections (A–C) × 9 periods. Figure 8a illustrates the geographical map of the target intersections. These densities varied with time. To analyze the characteristics of the densities with time, we introduced two densities: *central density* and *system density*. The former is defined as the number of nodes within 100 m of the center point; the range was within the time-to-crash of approximately 3 s at the relative speed of 120 km/h. As the central density is large, $d_{ir}$ in Equation (4) is, on average, small. System density represents the number of nodes within 300 m. As the density is large, |ℐ| in Equation (3) is also, on average, large. Figure 8b provides the average densities of each period. The trends of the system density and central density are proportional to the size of the intersections. Additionally, the system density is at its peak at 3500 s, as compared to the other times. The highest central densities were 3000 s at A, 1000 s at B, and 3500 s at C. In these periods, the performance is likely to be degraded.

### 4.3. Wireless Parameters

The key simulation parameters are listed in Table 2. First, the communication system model complied with the mode 4 standard [7]. A carrier frequency of 5.9 GHz was used as standard. The bandwidth was 10 MHz. The frame size was set to 190 bytes. We assumed that each frame conveyed accurate data that were sufficient to provide a warning. Additionally, we simply modeled the sub-channel of mode 4. To guarantee enough resources to transmit a frame of 190 bytes, mode 4 can have two sub-channels. Mode 4 has 1000 sub-frames/s at standard, and thus, the total number of slots was 2000 slots/s. Also, all the nodes were synchronized with GNSS.

Second, transmitted frames were correctly decoded when the SINRs were equal to or more than the threshold, as in [24,25]. The threshold was set to 5 dB [5]. As for SINR, radio propagation complied with the WINNER+ model [26], as recommended by METIS [27]. We used the LOS model to investigate the fundamental model and did not bind the specific NLOS environments. The propagation loss included a log-normal shadowing loss with a deviation of 3 dB, as established in [7]. We calculated the independent and identically distributed (i.i.d) shadowing loss between nodes. In the propagation model, the antenna height of all the nodes was 1.5 m, as was used in [13]. The noise power was set to −110 dBm [14].

Third, sensing-based SPS was configured as follows. The initial reselection counter was calculated at random, but within a range. The range was configured so that it continued to use a slot within 0.5 s–1.5 s for NTS $n_{t}$; for example, at 10 frames/s, the range was from 5 to 15. The standard determined the NTSs of 10, 20, and 50 frames/s. The ranges except for the standard NTS (from $R_{lower}$ to $R_{upper}$), were set to linear values, based on the range of 10 frames/s, as in Equation (8):

(8) $$\;\{\begin{array}{c} R_{upper}=\lfloor n_{t}\times 1.5\rfloor \\ R_{lower}=\lfloor n_{t}\times 0.5\rfloor \end{array}\;$$

The initial RSRP threshold was set to −110 dBm, which was the same as the noise power. The resource keeping probability was set to zero because of the most fundamental setting [7]. In other words, the nodes always reselected the next slot when the counter became zero.

## 5. Number of Received Frames and NAC in Two Crash Scenarios

This section presents the performance characteristics of mode 4 in crowded intersection crash scenarios and high-speed crash scenarios.

### 5.1. Crowded Intersection Scenarios

#### 5.1.1. Uniform Node Distribution Models

**Figure 9:**
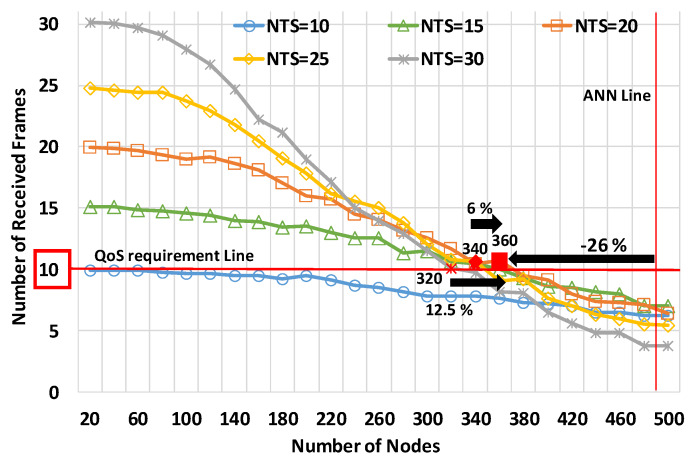
The number of received frames for the number of nodes in the uniform node distribution model at a relative speed of 120 km/h.

Figure 9 shows the number of received frames for the number of nodes at the relative speed of 120 km/h. The horizontal axis is the number of nodes, and the vertical axis is the number of received frames. The legends in Figure 9 represent the NTSs. In the evaluations, all the nodes were uniformly distributed.

First, we explain the performance characteristics of the NTSs. In particular, at the NTS of 30 frames/s, we can observe the performance trend, which is explained in Section 3.3. At the NTS, the number of received frames was the largest until reaching 220 nodes because of the redundant transmissions. However, it was the lowest at 500 nodes due to overloading frames. The performance sharply drops as the traffic load increases. In contrast, at the NTS of 15 frame/s, the number of received frames was the largest at 420 nodes, although it was the second lowest at 300 nodes. Therefore, configurating the NTS depends on the degree of the congestion situation.

In terms of CWS, Figure 9 demonstrates that mode 4, on average, provides less performance than the QoS requirements. In Figure 9, using the NTS of 20 frames/s provided the best performance of the five NTSs; specifically, the NAC was 26% less than the ANN (i.e., 488 nodes). The NAC at the NTS exceeded the NAC at the other NTSs, like 15 frames/s or 25 frames/s, by 6%. It was also 12.5% above the NAC at the NTS of 30 frames/s. Therefore, we highlighted that mode 4 showed the bestperformance at the NTS of 20 frames/s but was not able to satisfy the requirements.

#### 5.1.2. Realistic Node Distribution Models

**Figure 10:**
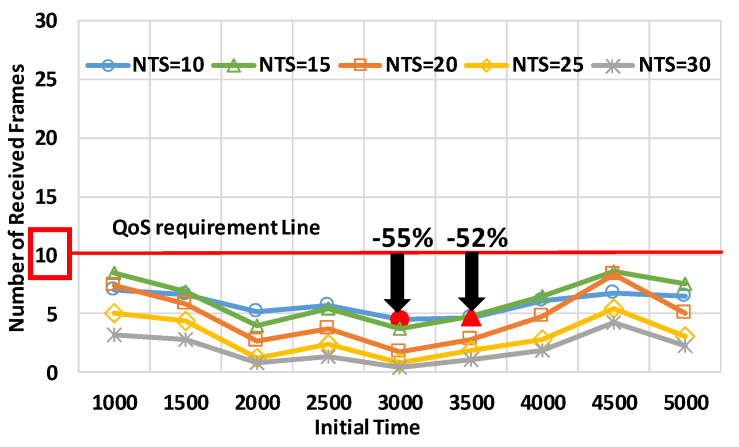
The number of received frames in the large intersection.

**Figure 11:**
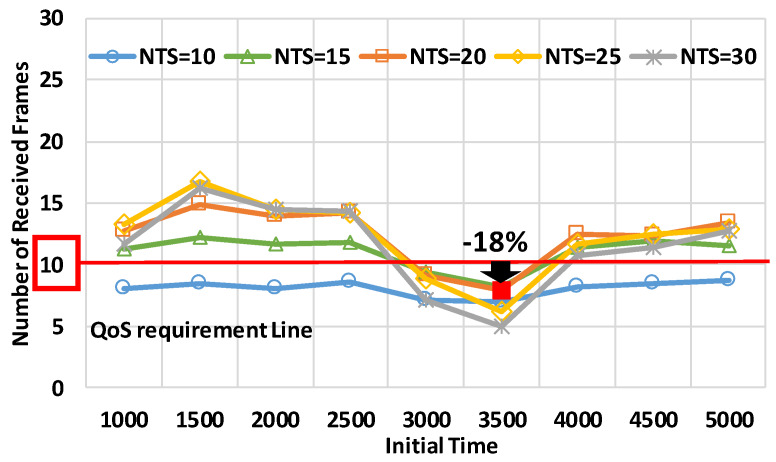
The number of received frames in the small intersection.

Figure 10 and Figure 11 depict the number of received frames in the large–small intersections at the five NTSs, respectively. The horizontal axes are the initial times of the periods of the traffic signal in each intersection. The vertical axes are the numbers of received frames. In the simulations, the relative speed of the target nodes was 120 km/h, which is to be assumed in urban areas.

Figure 10 demonstrates that the number of received frames did not satisfy the CWS requirements at any initial time in the large intersection at any NTS. From the results, using 10 frames/s and 15 frames/s showed better performance than performance at the other NTSs because the scenarios had many nodes, as compared to the other scenarios. At the initial time of 3000 s, using the NTS of 10 frames/s provided the best performance, and the number of received frames was 55% less than the QoS requirements. At the initial time of 3500 s, the suitable NTS was 15 frames/s, and the number of received frames was 52% less than the QoS requirements. The results emphasized that mode 4 failed to satisfy the requirements in the large intersection.

**Figure 12:**
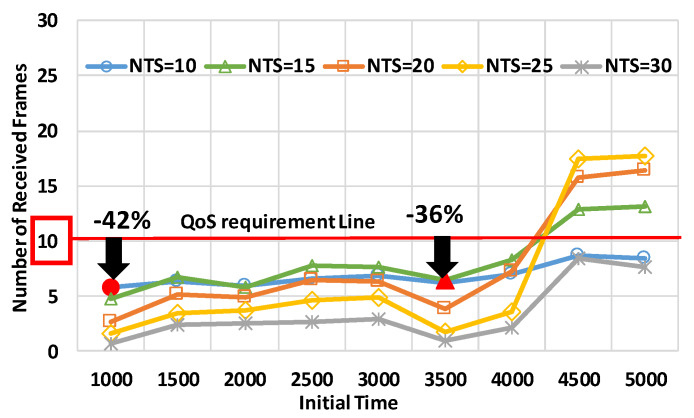
The number of received frames in the medium intersection.

Figure 12 shows that mode 4 failed to satisfy the QoS requirements during many periods in the medium intersection. In particular, the performance at the initial time of 1000 s or 3500 s was lower than the performance at the other periods. In the former period, mode 4 suppressed 42% fewer numbers of received frames, as compared to the QoS requirements. In the latter period, using the NTS of 15 frames/s provided the best communication quality, but the number of received frames was 36% less than the QoS requirements. At the periods of the initial time of 4500 or 5000, mode 4 showed enough performance to satisfy the requirements at the NTSs of 15, 20, and 25 frames/s.

As observed in Figure 11, the target node satisfied the QoS requirements at many periods at the small intersection. At periods except for the periods of the initial time of 3000 s or 3500 s, the number of received frames exceeded 10 frames/s. At the initial time of 3500 s or 3000 s, the target node did not receive a smaller number of frames than 10 frames at any NTS. In particular, at the initial time of 3500 s, the optimal NTS was 15 frames/s, and the number of received frames was 18% less than the QoS requirements. Therefore, we showed that mode 4 needed to improve the performance even in the medium and small intersections.

### 5.2. High-Speed Scenarios

**Figure 13:**
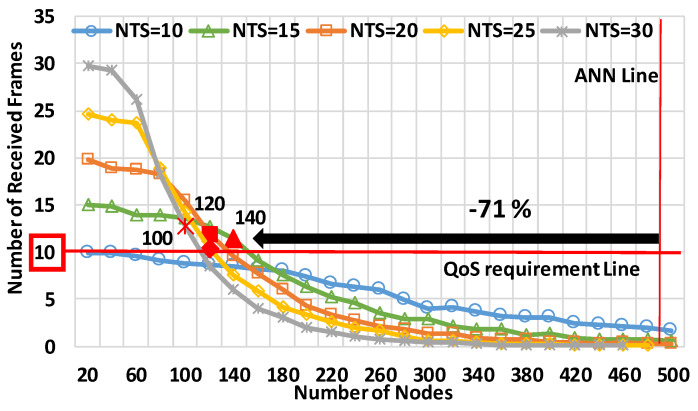
The number of received frames for the number of nodes at the relative speed of 240 km/h in the uniform node distribution model.

**Figure 14:**
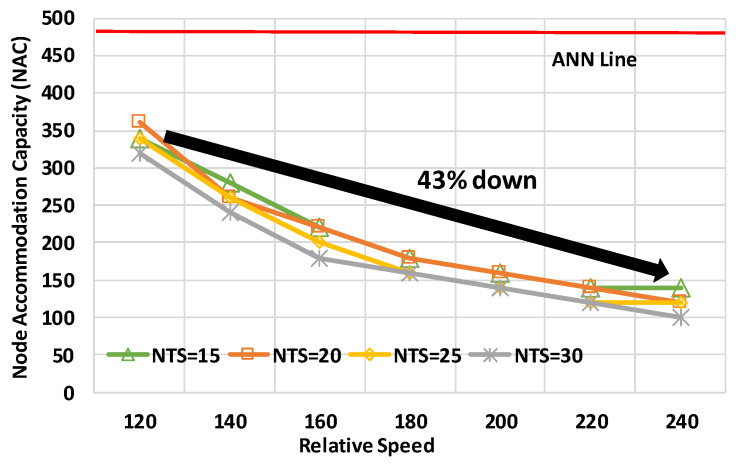
NACs for relative speed in the uniform distribution model.

Figure 13 and Figure 14 depict the performance characteristics in high-speed scenarios. Figure 13 shows the number of received frames for the number of nodes at a relative speed of 240 km/h. The horizontal axis, vertical axis, and legends are the same as in Figure 9. Figure 14 provides NAC characteristics for the relative speed between the target nodes. The horizontal axis is the relative speed, and the vertical axis is NAC. These results were evaluated in the uniform node distribution model to observe the average performance.

The two graphs highlight that the performance of mode 4 was sharply downgraded in high-speed scenarios. At first, Figure 13 shows that the NAC was significantly less than the ANN. The most effective NTS for NAC was 15 frames/s, and the NAC was, at most, 140 nodes. The resultant NAC was 71% less than ANN. After the number of nodes was 160, the best NTS was 10 frames/s, but the performance was much below the QoS requirements. Therefore, high-speed scenarios were provided to decrease the performance. Figure 14 demonstrates that the performance sharply dropped as the relative speed increased. At the NTS of 15 frames/s, the NAC at 240 km/h was 43% below that at 120 km/h. Therefore, concerning high-speed nodes, like emergency vehicles, it is necessary to significantly improve the performance of mode 4 for effective crash warning.

## 6. Conclusions

This paper investigated the feasibility of mode 4 in modeled crash scenarios in terms of performance under congestion conditions. The evaluation results highlight that mode 4 failed to satisfy the QoS requirements in many crash scenarios. At first, in crowded intersection scenarios, mode 4 provided low-reliable communications. In the uniform node distribution model, which evaluated average performance, the results demonstrated that NAC was 26% lower than the ANN. As well as the average performance, we evaluated more practical performance in the realistic node distribution model built by Bologna data. In the realistic large intersection, the number of received frames was 55% smaller than the QoS requirements. The high-speed scenarios, like emergency cars in urban areas, also cause congestion. When nodes collided with each other at a relative speed of 240 km/h, the NAC was 71% below the ANN. Our feasibility evaluations highlight the necessity to improve communication performance of mode 4 by new mechanisms like Decentralized Congestion Control to use CWS in the real world.

## Figures and Tables

**Table 1:** Summary of related works in terms of evaluated layers.

Layer		Crash Scenarios	Related Works
**PHY and MAC Layer**		Not assumed	[7,8,9,10,11,12,13]
Application Layer	Awareness		[14,15]
	Platoon		[16]
	CWS	Not assumed	Our previous works [17,18,19]
		Assumed	This work

**Table 2:** Key parameters in simulations.

Crash Scenario Parameters.	Values
NTS	10–30 frames/s
Node distribution model	Uniform, Realistic
Relative speed	120 km/h–240 km/h
**Wireless Settings**	**Values**
Carrier frequency	5.9 GHz
Bandwidth	10 MHz
Frame size	190 bytes
Radio propagation model	WINNER+ (LOS) B1
Shadowing deviation	3 dB (i.i.d)
Noise power	−110 dBm
SINR threshold	5 dB
**Configuration Settings**	**Values**
Reselection counter range	Linear Average
Initial RSRP threshold	−110 dBm
Resource keep probability	0

## References

[1] Xiong Z., Sheng H., Rong W., Cooper D.E. (2012). Intelligent transportation systems for smart cities: A progress review. Sci. China Inf. Sci..

[2] ElBatt T., Goel S.K., Holland G., Krishnan H., Parikh J. Cooperative collision warning using dedicated short range wireless communications. Proceedings of the 3rd International Workshop on Vehicular Ad Hoc Networks.

[3] U.S. Department of Transportation Announces Decision to Move Forward with Vehicle-to-Vehicle Communication Technology for Light Vehicles. https://www.auto-talks.com/u-s-department-transportation-announces-decision-move-forward-vehicle-vehicle-communication-technology-light-vehicles/.

[4] Bezzina D. Light Vehicle Platform Update. Proceedings of the IVBSS 2008 Public Meeting.

[5] Hirai T. (2019). Node Clustering Communication Method with Member Data Estimation to Improve QoS of V2X Communications for Driving Assistance with Crash Warning. IEEE Access.

[6] 3GPP (2017). Evolved Universal Terrestrial Radio Access (E-UTRA).

[7] Molina-Masegosa R., Gozalvez J. (2017). LTE-V for Sidelink 5G V2X Vehicular Communications: A New 5G Technology for Short-Range Vehicle-to-Everything Communications. IEEE Veh. Technol. Mag..

[8] Bazzi A., Member S. (2018). Study of the Impact of PHY and MAC Parameters in 3GPP C-V2V Mode 4. IEEE Access.

[9] Gonzalez-Martin M., Sepulcre M., Molina-Masegosa R., Gozalvez J. (2018). Analytical Models of the Performance of C-V2X Mode 4 Vehicular Communications. IEEE Trans. Veh. Technol..

[10] Park Y., Weon S., Hwang I., Lee H., Kim J., Member S. Spatial Capacity of LTE-based V2V Communication. Proceedings of the 2018 International Conference on Electronics, Information, and Communication (ICEIC).

[11] Nabil A., Marojevic V., Kaur K., Dietrich C. (2018). Performance Analysis of Sensing-Based Semi-Persistent Scheduling in C-V2X Networks. arXiv.

[12] Toghi B., Mughal M.O., Mahjoub H.N., Fallah Y.P., Rao J., Das S. (2018). Multiple Access in Cellular V2X: Performance Analysis in Highly Congested Vehicular Networks. arXiv.

[13] He J., Tang Z., Fan Z., Zhang J. (2018). Enhanced collision avoidance for distributed LTE vehicle to vehicle broadcast communications. IEEE Commun. Lett..

[14] Cecchini G., Bazzi A., Masini B.M., Zanella A. Performance comparison between IEEE 802.11p and LTE-V2V in-coverage and out-of-coverage for cooperative awareness. Proceedings of the 2017 IEEE Vehicular Networking Conference (VNC).

[15] Bazzi A., Masini B.M., Zanella A. How many vehicles in the LTE-V2V awareness range with half or full duplex radios?. Proceedings of the 2017 15th International Conference on ITS Telecommunications (ITST).

[16] Vukadinovic V., Bakowski K., Marsch P., Garcia I.D., Xu H., Sybis M., Sroka P., Wesolowski K., Lister D., Thibault I. (2018). 3GPP C-V2X and IEEE 802.11p for Vehicle-to-Vehicle Communications in Highway Platooning Scenarios. Ad Hoc Netw..

[17] Takeshi H., Tutomu M. NOMA Concept for PC5-based Cellular-V2X mode 4 in Crash Warning System. Proceedings of the 2019 IEEE 90th Vehicular Technology Conference.

[18] Takeshi H., Tutomu M. Performance Characteristics of Sensing-based SPS of PC5-based C-V2X Mode 4 in Crash Warning Application under Congestion. Proceedings of the IEEE ITSC 2019.

[19] Takeshi H., Tutomu M. Practical Performance of Simple C-V2X Mode 4 using NOMA for Driver Assistant System with Crash Warning. Proceedings of the 6th International Workshop on Smart Wireless Communications.

[20] Kawasaki R., Onishi H., Murase T. Performance evaluation on V2X communication with PC5-based and Uu-based LTE in Crash Warning Application. Proceedings of the IEEE GCCE 2017.

[21] Bieker L., Krajzewicz D., Morra A.P., Michelacci C., Cartolano F. (2015). Traffic simulation for all: A real world traffic scenario from the city of Bologna. Lect. Notes Control Inf. Sci..

[22] Bedogni L., Gramaglia M., Vesco A., Fiore M., Härri J., Ferrero F. (2015). The Bologna ringway dataset: Improving road network conversion in SUMO and validating urban mobility via navigation services. IEEE Trans. Veh. Technol..

[23] Behrisch M., Bieker L., Erdmann J., Krajzewicz D. SUMO–Simulation of Urban Mobility. Proceedings of the SIMUL 2011, The Third International Conference on Advances in System Simulation.

[24] Hadzi-Velkov Z., Spasenovski B. Capture Effect in IEEE 802.11 Basic Service Area under Influence of Rayleigh Fading and Near/Far Effect. Proceedings of the 13th IEEE International Symposium on Personal, Indoor and Mobile Radio Communications.

[25] Li X., Zeng Q.-A. Capture effect in the IEEE 802.11 WLANs with rayleigh fading, shadowing, and path loss. Proceedings of the 2006 IEEE International Conference on Wireless and Mobile Computing, Networking and Communications.

[26] Juha M., Pekka K., Lassi H., Tommi J., Essi S., Esa K. (2010). Milan, D5.3: WINNER+ Final Channel Models, Wireless World Initiative New Radio WINNER. https://www.yumpu.com/en/document/view/37612823/d53-winner-final-channel-models-celtic-plus.

[27] METIS EU Project Consortium (2014). Initial Channel Models Based on Measurements. https://metis2020.com/.

